# Corrections to “Sparse Multichannel Decomposition of Electrodermal Activity With Physiological Priors”

**DOI:** 10.1109/OJEMB.2024.3444428

**Published:** 2024-08-26

**Authors:** Samiul Alam, Md. Rafiul Amin, Rose T. Faghih

**Affiliations:** Department of Electrical and Computer EngineeringUniversity of Houston 14743 Houston TX 77004 USA; Department of Electrical and Computer EngineeringUniversity of Houston 14743 Houston TX 77004 USA; Department of Biomedical EngineeringNew York University 5894 New York NY 10010 USA

## Abstract

Presents corrections to the article “Sparse Multichannel Decomposition of Electrodermal Activity With Physiological Priors”.

The article “Sparse Multichannel Decomposition of Electrodermal Activity With Physiological Priors” [1] published in IEEE Open Journal of Engineering in Medicine and Biology in 2023, describes a way to concurrently decompose electrodermal activity (EDA) from multichannel recordings. In it, the continuous form state space equation for $m$ channel EDA, given by (9) and (10), contains a typo. The correct equations will be:

\begin{align*}
{\dot{x}_{2m - 1}}{\left( t \right)}&=- \frac{1}{{{{\tau }_{{{r}_m}}}}}{{x}_{2m - 1}}\left( t \right) + \frac{1}{{{{\tau }_{{{r}_m}}}}}u\left( t \right),\\
{\dot{x}_{2m}}\left( t \right)&= \frac{1}{{{{\tau }_{{{d}_m}}}}}{{x}_{2m - 1}}\left( t \right) - \frac{1}{{{{\tau }_{{{d}_m}}}}}{{x}_{2m}}\left( t \right).
\end{align*}
